# The art of modeling gene regulatory circuits

**DOI:** 10.1038/s41540-024-00380-2

**Published:** 2024-05-29

**Authors:** Mariana Gómez-Schiavon, Isabel Montejano-Montelongo, F. Sophia Orozco-Ruiz, Cristina Sotomayor-Vivas

**Affiliations:** 1 International Laboratory for Human Genome Research, Universidad Nacional Autónoma de México, Queretaro, 76230 Mexico; 2 ANID—Millennium Science Initiative Program—Millennium Institute for Integrative Biology (iBio), Santiago, 8331150 Chile

**Keywords:** Regulatory networks, Computer modelling

## Abstract

The amazing complexity of gene regulatory circuits, and biological systems in general, makes mathematical modeling an essential tool to frame and develop our understanding of their properties. Here, we present some fundamental considerations to develop and analyze a model of a gene regulatory circuit of interest, either representing a natural, synthetic, or theoretical system. A mathematical model allows us to effectively evaluate the logical implications of our hypotheses. Using our models to systematically perform in silico experiments, we can then propose specific follow-up assessments of the biological system as well as to reformulate the original assumptions, enriching both our knowledge and our understanding of the system. We want to invite the community working on different aspects of gene regulatory circuits to explore the power and benefits of mathematical modeling in their system.

## Introduction

Gene regulation is fundamental in any biological process, and we must understand its underpinnings to make sense of the amazing complexity of life. It is through gene regulation that organisms modulate the activity of their constitutive processes, such as metabolism, cell cycle, etc., in a given context. In other words, it translates the information encoded in a genotype to a specific phenotype depending on the cellular environment. Not surprisingly, gene regulation is studied from multiple perspectives, ranging from characterizing specific regulatory sequences to reconstructing regulatory networks via large scale transcriptional profiles^1^. In order to fully understand gene regulation, describing individual steps is insufficient, we need to determine how information flows through these regulatory steps and their dynamic properties. A network representation of this information flow is commonly known as a gene regulatory circuit, an analogy dating back to the early 60s^2^, which has gained popularity with the rise of systems biology and synthetic biology.

A gene regulatory circuit is a representation of the interactions between the entities involved in a system of interest, and the computing logic of such interactions. In different scenarios, the specifics of these gene regulatory circuits might vary; for example, the links between entities might represent molecule-to-molecule interactions, or they might represent correlations derived from the data without explicit knowledge of the underlying mechanism. Studying their structural principles and dynamic properties has been the focus of the fields of systems biology and synthetic biology over the last two decades (e.g., see ref. ^3^). These studies have helped us to understand the fundamentals of life (e.g., cell cycle regulation^4,5^; cell differentiation^6^; cellular interactions with the environment and other cells^7,8^) to develop important applications in biotechnology (e.g., enhancing a plant’s response to parasites^9,10^; improving the yield of microbial factories^11^; or even engineering bacterial structures capable of sensing pressure^12^), and to facilitate advances in medicine (e.g., unveiling mechanisms relevant for disease progression^13^).

An essential tool to study gene regulatory circuits is mathematical modeling. Gene regulatory circuits are dynamic systems, often involving nonlinear and saturable functions, as well as feedforward and feedback loops^14,15^. Together, these give rise to emergent properties that cannot be explained by looking at the individual parts in isolation. These emergent properties are often nontrivial, constantly test our intuition about the system, and make the mathematical approach indispensable to effectively evaluate them. Noteworthy, these properties are not exclusive of gene regulatory circuits. Many biological systems, over wide range of temporal and structural scales, are also nonlinear dynamic systems (e.g., signalling pathways, physiological responses, and ecological interactions). Consequently, many of the tools used for modeling gene regulatory circuits –and many of the points discussed here– apply as well for these other systems. Here we focus on gene regulatory circuits, but we advice the reader to keep an open mind about what the elements in the circuits could be representing other than genes.

In reality, building mathematical models of gene regulatory circuits is as much an art as it is a technique. Through our collective experiences and insights, we aim to foster a deeper appreciation for the intricate balance between creativity and methodology inherent in the construction of mathematical models. Here, we discuss some fundamental considerations to develop a model of any gene regulatory circuit of interest, either representing a natural system to be better understood, a synthetic system to be characterized in the lab, or a theoretical system to learn more about its emergent properties per se. These points summarize on one side our experience as young students diving into modeling biological systems for the first time, and on the other side our insights after teaching this topic to diverse audiences –in the United States and Latin America, from undergrad students to faculty in diverse fields. Maybe even more importantly, we write these discussion points as true enthusiasts of mathematical modeling of gene regulatory circuits, as we hope to see more of these models in the literature, and attract more young scientists to study their dynamic properties.

## Think of your model as a logical machine

We actually build models in biology –and science in general– all the time. Understanding requires an abstraction, and this abstraction requires building a model of the complex reality we aim to understand. In that sense, all good experiments are also good abstractions which require good modeling^16^. Similarly, mathematical modeling is actually just one type of experimental design: assumptions represent our previous knowledge about the system and the proposed hypothesis; mathematical equations are our experimental model; and the quantitative predictions of such equations equate to our observations used to evaluate and –hopefully– improve our initial hypothesis.

In fact, models are simply scientific devices that allow us to articulate the expectations of a specific theory or hypothesis^17^. How much the model’s predictions relate to the observations from the natural system can only show how accurate the underlying theory is, but the model cannot be per se incorrect (excluding of course trivial –and sometimes nontrivial– technical mistakes). As such, a model should be understood as a logical machine to derive the implications of our previous belief or knowledge^18^, nothing more and nothing less. And when talking about logic, mathematical language is naturally the ideal framework. As nicely put by Strogatz^19^: “[mathematics] is much more than a language; it’s also an incredibly powerful system of reasoning. […] The symbol shuffling is useful shorthand, a convenient way to build arguments too intricate to hold in our heads.”

## Know your system

A model is useful only when it effectively reflects the hypothesis to be tested. For this reason, an essential point here, which cannot be emphasized enough, is: know your system! John von Neumann is often credited with the phrase “with four parameters I can fit an elephant, and with five I can make him wiggle his trunk”^20^. This quote reminds us that, with enough free parameters, we can always get a mathematical model to fit our data. That does not mean that the model is informative. Our model must always be derived from the abstraction of the available knowledge about our system, and be grounded on the specific hypothesis to be tested.

In the case of gene regulatory circuits, we need to determine what is known about the relevant molecules involved in the system to be modeled (e.g., transcription factors), as well as their interaction mechanisms and outcomes (e.g., transcriptional repression). Biochemical details about the mechanism of each interaction are always helpful to inform our model, but the amount of information explicitly included in the model will depend on the question and purpose of the model (further discussed below). Additionally, we should consider the specific data that will inform the model (e.g., single-cell *vs* population measurements), understanding their properties, limitations, and potential biases.

## Set down your assumptions, all of them

As discussed above, the assumptions are precisely what we aim to test using our model as a logical machine. Nevertheless, very often researchers erroneously presume these assumptions as obvious, leaving them out of the explanation of their model’s construction and, more importantly, the interpretation of their results. Both as good practice and to fully take advantage of our model, we must explicitly set down all of our assumptions. Of note, during the abstraction process we often rely on simplifying assumptions that we know are essentially false in most cases (e.g., assuming that the studied circuit is acting in isolation, while in reality biological molecules are highly promiscuous^21^). Being aware of these approximations and potential problems will help us to better judge our model’s behavior and the extent of the validity of our results.

## Choose your question and decide how to approach it

In parallel with formulating our model assumptions, we must carefully define the question to be answered, always considering the available data and means. Each model has different advantages and limitations, as well as different requirements for what should inform them. In particular, we need to judiciously decide the level of granularity or detail to be included in our model. As Rosenblueth & Wiener^16^ ironically said: “The best [...] model of a cat is another, or preferably the same cat”. Basically, attempting to include all the details of the original system in a model would be both unnecessary and fruitless, as we could simply use “the actual cat”, and often these details are unknown. Remember, the goal of a model is to build an abstraction of your previous knowledge, making it more manageable and enabling you to actually comprehend the studied system. You need to evaluate both what is expected to be relevant in your system, and how such processes can be informed. Importantly, keep in mind that the complexity of the question will determine the type of model required. For instance, a simple differential equation model may suffice to describe the bulk dynamics of your system, while an agent-based model could be more appropriate for capturing spatial emergent properties. Additionally, depending on your research question, a simple or small-scale model may be appropriate for delineating general biological principles, while larger-scale models with higher detail allow for more accurate quantitative predictions (see ref. ^22^ & ref. ^23^ for further discussion).

## Define your circuit

**Fig. 1:**
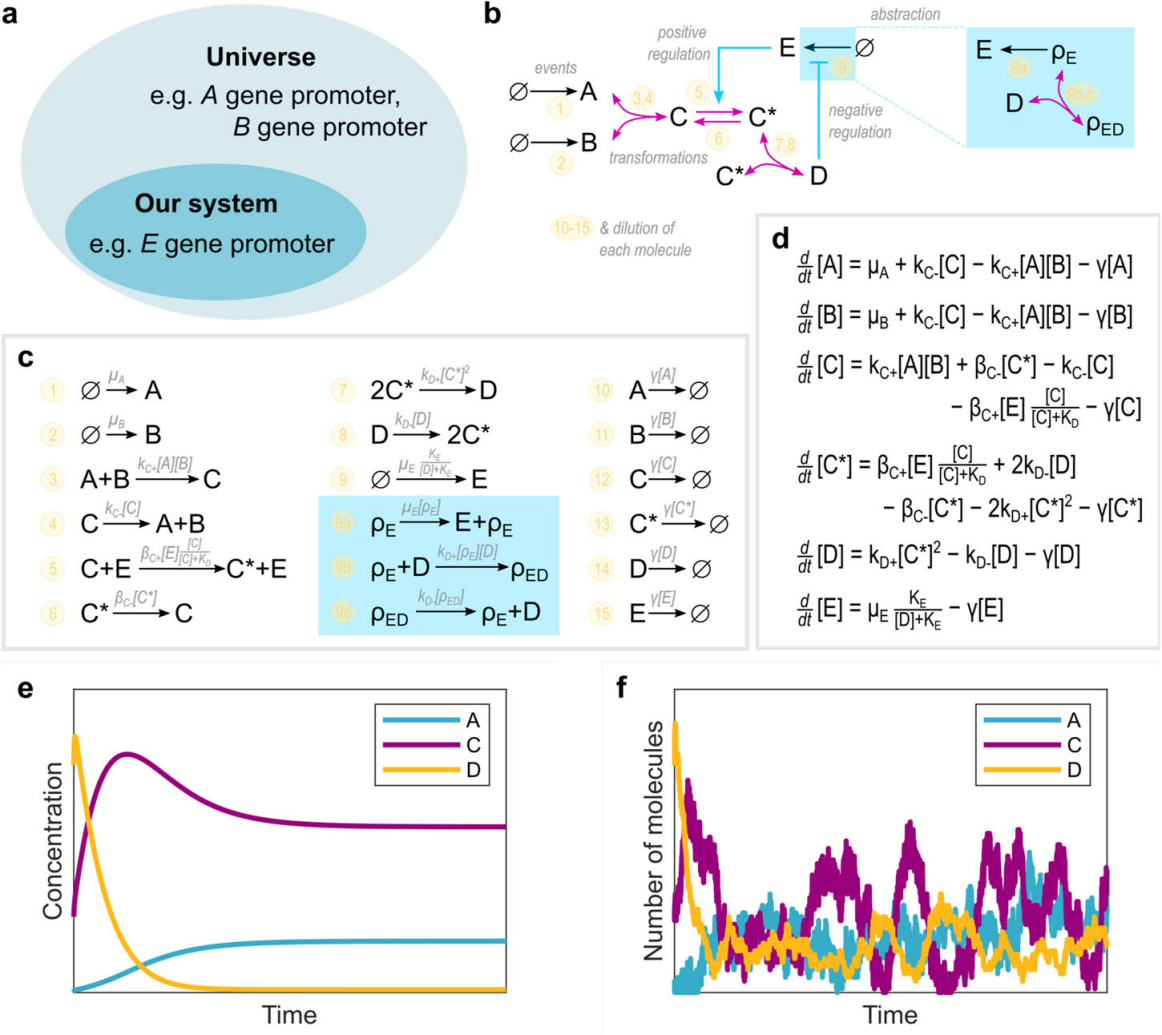
Exemplifying the steps for modeling a gene regulatory circuit. Depending on the available information and our specific question, we define our gene regulatory circuit by first (**a**) delineating what is to be explicitly considered in our model –our system– and what is not –the universe–, and second (**b**) by representing our system as a circuit, i.e., the set of entities or molecular species and the interactions between them. The example presented here –for didactic purposes– highlights how different types of interactions might be represented: biochemical events (black arrows; e.g., synthesis of a protein), transformations –where the entities are consumed (pink arrows; e.g., binding/unbinding or phosphorylation of proteins), and regulation of biochemical reactions or events, either positive regulation –facilitating it (blue arrows; e.g., an enzyme), or negative regulation –obstructing it (blue blunt arrows; e.g., a transcriptional repressor). Also, some abstractions or approximations considered in the model can be represented in our circuit diagram, e.g., by condensing the multiple biochemical reactions needed for protein synthesis into a single event, or by disregarding the binding of a transcriptional factor to the promoter as part of its regulation. Then, following the map presented by our circuit diagram, we can write down (**c**) the reaction equations describing the relevant biochemical events and (**d**) the associated ordinary differential equations. Finally, for some given biochemical parameter values and starting conditions, we can explore the gene regulatory circuit dynamics, either (**e**) deterministically or (**f**) stochastically.

Defining our circuit is the first step to actually building our mathematical model of the gene regulatory system of interest. The proposed circuit must recapitulate what we know about our system and our assumptions, as well as conforming with our specific question. The visual representation of our circuit accomplishes two important goals: Firstly, the circuit represents our abstraction of the system to be studied from the entirety of the complex reality, delineating the border between “our system” –what is explicitly in the circuit– and the “universe” –the rest (Fig. 1a). Secondly, the circuit provides a map for building our mathematical model, where each node represents an entity or molecular species (e.g., a relevant transcription factor) and each edge denotes an interaction or biochemical reaction to consider (e.g., activating a gene promoter; Fig. 1b). Each of these elements will then be included in our model equations. Moreover, this map facilitates the identification of functional motifs within the regulatory circuit. A motif, defined as a simple pattern among a small number of interacting entities^24^, plays a crucial role in determining dynamic behaviors. For instance, negative feedback is essential for oscillatory behavior, while positive feedback is necessary for displaying multistability^14^. Recognizing these motifs helps us understand the potential dynamic properties of our system. However, it is important to note that the motifs present in our map only provide insights into what is theoretically possible in our proposed model. It is possible that crucial interactions for emergent properties were not considered explicitly in our model, or that non-explicit feedback mechanisms contribute to emergent properties (e.g., see refs. ^25^,^26^).

## Write down the biochemical events

Following the map created by defining our circuit, we need to write down explicitly all the biochemical events that occur in our model. Here, the definition of an “event” will be directly linked to the level of abstraction chosen; e.g., a biochemical event might simply be the synthesis of our protein of interest, or transcription and translation might be considered as two biochemical events. A useful way to represent these biochemical events is as reaction equations, where the “reactants” to be consumed are put on the left of the equation, the resulting “products” are put on the right, and the arrow in the middle depicts the “flux function” associated with the reaction rate (Fig. 1c). We often include molecules that are crucial for a reaction, but not consumed, in both sides of a reaction equation to explicitly communicate this dependency (e.g., the mRNA in a translation event). This set of equations defines how our system changes over time, and exactly which molecules are affected. Of note, formulating the reaction flux functions to truly reflect the underlying biochemical mechanisms is one of the most important aspects of our model. We need to once again pay particular attention to what is known about our system and to our specific assumptions. Keep in mind that some of the events in our model might correspond to molecule-molecule interactions following the mass action law, or they might involve multiple or complex molecular interactions better represented by phenomenological functions, such as Michaelis-Menten or Hill-like functions^27^. We might have to revisit our assumptions to clearly incorporate any approximation used for the reaction flux functions in our model.

## Locate your system in the parameter space

All gene circuits are fundamentally dynamic. Their dynamics are described by the “flux functions” or rates associated with each biochemical event. These functions in turn will depend on the values of their parameters, which reflect both the biochemical context (e.g., temperature, availability of resources, etc.) and the molecular properties of the involved molecules (e.g., protein-protein affinity, enzymatic catalytic rates). Measuring most of these parameters in vivo can be challenging. The few available measurements tend to be unreliable, varying not only with the very specific details of the experimental setting, but also among day-to-day replicas of the same experiment. This is expected given the dependency of the parameters on the biochemical context, which is by nature variable. Luckily, many of the relevant properties of our system can be inferred simply by locating it in the parameter space, without assigning a unique fixed value to your parameters.

A way to achieve this is through parameter fitting, by systematically exploring which parameter values effectively reproduce the desired (observed or theoretical) behavior of our system (ideally through Bayesian approaches, but that discussion lies outside the scope of this manuscript; see ref. ^28^). Note that experimental measurements of relevant molecule half-lives can be extremely helpful as they fix the time scale of the model and restrict possible fitting solutions. This highlights another crucial issue: parameter identifiability. In a model, a set of parameters is considered unidentifiable if multiple sets of parameter values result in indistinguishable behaviors^29^. Being aware of parameter identifiability in your model is essential for effective parameter fitting and result interpretation. Another useful approach is sensitivity analysis, which measures the effect of small changes in parameter values on model behavior^30^. Through sensitivity analysis, we can gain insights into the most relevant parts of our circuit, which events contribute more to specific properties, and evaluate the robustness or potential fine-tuning of our model.

A great alternative to parameter fitting is what is called phenotype-centric modeling, developed by Savageau and colleagues^31^, which allows us to characterize the whole parameter space into regions with specific phenotypes or qualitative behaviors. For either of these approaches, we will need to solve or simulate the response of our model. The specifics to take in account for this are discussed in the following two points.

## Get an intuition for the shape of your system

Diverse mathematical frameworks offer avenues to explore the dynamic properties of our system. The choice of which one to use depends strongly on our specific question, with three crucial decisions to make: deterministic or stochastic dynamics, continuous or discrete time, and continuous or discrete species (see ref. ^32^). A subsequent section will discuss the rationale behind modeling stochastic dynamics. Boolean networks are suitable for modeling discrete states and time, ideal for qualitatively exploring systems with numerous components and limited kinetic information (see ref. ^33^). When more kinetic data is available, continuous-time modeling using differential equations prevails, offering rich analysis and simulation tools.

Constructing these differential equations is straightforward with the list of reaction equations for our model: For each molecular species in our model (*X*_*i*_), its rate change function (*dX*_*i*_/*dt*) will be the sum of all the flux functions of the reactions equations where the molecular species is a product (i.e., present at the right side of the reaction equation) minus the sum of all the flux functions of the reactions equations where the molecular species is a reactant (i.e., present at the left side of the reaction equation), multiplied in each case by the number of *X*_*i*_ molecules produced or used, respectively, in the reaction (Fig. 1d).

While analytical solutions are rare, basic approaches yield valuable insights. Steady state values (*dX*_*i*_/*dt* = 0) and stability can be easily determined (e.g., whether the system converges to a steady state or oscillates; see ref. ^34^), and bifurcation analysis assesses how steady states change or appear and disappear with parameter variations (see ref. ^34^). Similarly, simulations from varied initial conditions offer behavioral insights (Fig. 1e) and aid parameter fitting. Differential equations also inform the phenotype-centric modeling approach.

## Deal with the unavoidable noise

As briefly discussed above, in the abstraction process we often rely on approximations that we know are essentially inaccurate. One of the most ubiquitous of these is modeling a biological system as a deterministic process (e.g., using differential equations). Molecular systems are inherently stochastic, making biochemical noise not only unavoidable, but an essential ingredient of many biological mechanisms (see refs. ^35–37^ for further discussion). This stochasticity arises from the discrete nature of molecules and the probabilistic nature of biochemical reactions (referred to as “intrinsic” noise), as well as from environmental factors (known as “extrinsic” noise), such as variation in resource availability or temperature fluctuations. For this reason, an important consideration for modeling gene regulatory circuits is to explore the effect of the inherent stochasticity on the system’s behavior. We recommend the following two alternative approaches for this. We can represent our system using the chemical master equation (CME), an infinite set of differential equations that show the change rates of the probability of your system being in each possible state. This might sound intimidating, but it is actually very intuitive and tractable. Each change rate equation can easily be defined using our list of reaction equations, considering all the reactions that take the system to and from the specific state and their probability of occurrence (flux functions representing the propensity of a reaction to occur given the system state multiplied by the current probability of being in such state). As the flux functions –now called propensities– evaluated in a specific state have a constant value, the CME set of differential equations is actually a linear transformation, making its analysis much easier than the (often) highly nonlinear differential equations representing the deterministic model^38,39^. Moreover, it has been shown that the CME has a unique solution (where the change on the probability of being in each possible state is equal to zero) and the system always converges to it (i.e., a stable solution)^27^, and also that the infinite set of equations (with the resulting infinite matrix representing the linear transformation) can be truncated with a guaranteed accuracy^40^. Alternatively, the popular Gillespie algorithm (or stochastic simulation algorithm, SSA) can be used to simulate the stochastic dynamics of our list of the reaction equations, once again with the flux functions representing the propensity of each reaction^41^ (Fig. 1f).

## And then? Use your model!

**Fig. 2:**
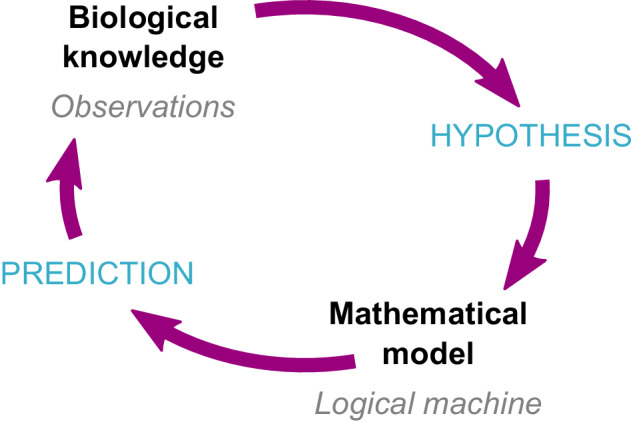
Feedback between biological knowledge and mathematical modeling. A specific hypothesis can be proposed from our observations of a biological system. The logical consequences of this hypothesis can then be evaluated through a mathematical model (i.e., logical machine). Using this model, we can make predictions to be tested with new experiments or observations to enrich our knowledge about the system.

While building a model of our system can be a very instructive (and fun) exercise, forcing us –at least while considering the points proposed here– to seriously think about our knowledge of the molecular mechanisms and their logical implications, it is rarely the final goal. By having the mathematical model logically representing our hypotheses about the system, we can efficiently and systematically carry out many different in silico experiments to evaluate our hypotheses and propose expected behaviors. For example, we can observe the range of responses as a stimulus is varied (e.g., ref. ^42^), test the sensitivity or robustness of our system to varying conditions or parameters (e.g., ref. ^43^), or predict the effect of specific mutants or epigenetic changes (e.g., ref. ^44^). The bulk of observations from these in silico experiments can then be used to propose specific follow-up assessments of the actual biological systems as well as to reformulate the original assumptions, enriching both our knowledge and our understanding of the system (Fig. 2).

## Conclusions

The remarkable complexity of gene regulatory circuits and biological systems necessitates the use of mathematical modeling as an indispensable tool to unravel their dynamic properties. While skepticism once prevailed in the biological community regarding the efficacy of mathematical models due to the intricate nature and vast unknowns of biological systems, history has proven otherwise. Mathematical modeling has actually long played a pivotal role in biology, from the emergence of population genetics in the early twentieth century to the enduring tradition in ecology (see ref. ^18^ for a captivating exploration of the historical significance and power of mathematical models in biology). In recent years, success stories in the realm of gene regulatory circuits have transformed the perception of mathematical modeling from skepticism to essentiality. One striking example is the beautiful work by Novak and Tyson^45^, whose mathematical model of the cell-cycle in yeast yielded predictions on mitotic control later validated through extensive experimental investigations (see ref. ^46^, for a complete recount of this story). Such achievements underscore the transformative impact of mathematical modeling in unraveling the complexities of gene regulatory networks and affirm its status as an indispensable approach in contemporary biological research.

Given this importance, we encourage readers to incorporate mathematical modeling into their scientific toolbox. The simple points discussed here give a step-by-step roadmap to develop and analyze a model of our favorite gene regulatory circuit. These are of course not exhaustive, as each individual system will include its own subtleties and complications. As mentioned in the introduction, in reality modeling is as much an art as it is a technique. But we want to invite the community working on different aspects of gene regulatory circuits to give it a try, to explore the power and benefits of mathematical modeling in their system, and to see where their artistry finally takes them.
